# Supplementary material for: Dual Molecular Diagnoses of Recessive Disorders in a Child from Consanguineous Parents: Case Report and Literature Review
Source: Genes (Basel). 2022 Dec 16;13(12):2377. doi: 10.3390/genes13122377 (PMC9778442; doi:10.3390/genes13122377)
Supplement: Supplementary file 1 [file genes-13-02377-s001.zip › genes-2089851-supplementary.pdf]

Supplementary Table S1. Multiple Pathogenic Variants in Patients with Autosomal Recessive Conditions

Dual Molecular Diagnoses (AR + AR)	Submitter	Consanguinity
AGL + PCCA	Posey et al, 2017 [1]	+
HEXB + MCCC2	Posey et al, 2017 [1]	+
MTPAP + NPC1	Posey et al, 2017 [1]	+
RECQL4 + XPC	Posey et al, 2017 [1]	+
TPO + MMP2	Posey et al, 2017 [1]	+
AGL + LPAR6	Posey et al, 2017 [1]	+
PAPSS2 + TRDN	Posey et al, 2017 [1]	+
OTOF + SLC12A6	Posey et al, 2017 [1]	+
AGPAT2 + OBSL1	Posey et al, 2017 [1]	+
MCCC1 + GNB5	Shao et al, 2021 [25]	+
UGT1A1 + ABCC2	L Slachtova et al, 2016 [26]	+
UGT1A1 + ABCC2	L Slachtova et al, 2016 [26]	+
UGT1A1 + ABCC2	L Slachtova et al, 2016 [26]	-
UGT1A1 + ABCC2	L Slachtova et al, 2016 [26]	-
PKHD1 + ATM	Balci et al, 2017 [3]	-
G6PC3 + SLC45A2	Balci et al, 2017 [3]	+
CYP26C1 + MED25	Balci et al, 2017 [3]	+
ERCC6 + BCKDHA	Balci et al, 2017 [3]	+
MAPRE2 + CDON	Berkun et al, 2019	+
HPSE2 + SMPD1	Cesur Baltacı et al, 2021 [28]	+
UGT1A1 + ABCC2	Cebecauerova et al, 2005 [29]	-
ATP7B + GALT	Doğulu et al, 2022 [30]	+
AMPD2 + AP4B1	Karaca et al, 2018 [10]	+
C12ORF65 + GPR126	Karaca et al, 2018 [10]	+
ITGA7 + MAGI3	Karaca et al, 2018 [10]	+
CUL7 + ILDR1	Akalin et al, 2022 [31]	+
NBEAL2 + DNAH5	Al-Dewik et al, 2019 [32]	-
GLE1 + ATM	Al-Dewik et al, 2019 [32]	-
LAMA2 + TMPRSS4	Al-Dewik et al, 2019 [32]	+
NEB + GALC	Al-Dewik et al, 2019 [32]	-
RPIA + LAMTOR4	Al-Dewik et al, 2019 [32]	-
KDM2B + RSPO4	Mitani et al, 2021 [12]	+
HECTD4 + TRAK1	Mitani et al, 2021 [12]	+
GOLGA2 + MRPS27	Mitani et al, 2021 [12]	+
CEP290 + SLC37A1	Mitani et al, 2021 [12]	+
SORCS2 + ACOT7	Mitani et al, 2021 [12]	+
DLGAP1 + OCLN	Mitani et al, 2021 [12]	+
TUBB6 + WDR81	Mitani et al, 2021 [12]	+
AMPD2 + MKS1	Mitani et al, 2021 [12]	+

DUSP4 + SLC39A10	Mitani et al, 2021 [12]	+
SSTR3 + TMOD1	Mitani et al, 2021 [12]	+
ADSL + TNRC6B	Mitani et al, 2021 [12]	+
LPAR6 + PLK4	Mitani et al, 2021 [12]	+
SSH3 + TRMT1	Mitani et al, 2021 [12]	+
LCTL + PLAA	Mitani et al, 2021 [12]	+
ASNS + RASGRF2	Mitani et al, 2021 [12]	+
FAM120A + STUB1	Mitani et al, 2021 [12]	+
COBL + DDC	Mitani et al, 2021 [12]	+
LRP2 + PCDH18	Mitani et al, 2021 [12]	+
GIT1 + WDR83OS	Mitani et al, 2021 [12]	+
ARV1+ ASH1L	Mitani et al, 2021 [12]	+
CNTNAP2 + STOML1	Mitani et al, 2021 [12]	+
CLP1 + CNTN5	Mitani et al, 2021 [12]	+
SCN7A + SPR	Mitani et al, 2021 [12]	+
FSHR + LAMA1	Mitani et al, 2021 [12]	+
NANS + NRD1	Mitani et al, 2021 [12]	+
PLD3 + RNASEH2A	Mitani et al, 2021 [12]	+
CIT + ULK2	Mitani et al, 2021 [12]	-
C2ORF69 + PARD3B	Mitani et al, 2021 [12]	-
G6PC + GAA	Monies et al, 2019 [33]	+
CRB1 + ADIPOR1	Monies et al, 2019 [33]	+
ECEL1 + FLII	Pehlivan et al, 2019 [11]	+
NEB + MID1IP1	Pehlivan et al, 2019 [11]	+
COG6 + MED27	Pehlivan et al, 2019 [11]	+
TNNT1 + PRODH	Smith et al, 2019 [4]	NA
ACAT1 + ETFB	Smith et al, 2019 [4]	NA
DNAH5 + TMEM67	Smith et al, 2019 [4]	NA
CUL7 + ACAN	Smith et al, 2019 [4]	NA
DOK7 + SACS	Smith et al, 2019 [4]	NA
TCTN2 + UNC45B	Smith et al, 2019 [4]	NA
AUH + SGSH	Smith et al, 2019 [4]	NA
RNASEH2A + PNPK	Smith et al, 2019 [4]	NA
CSTB + CEP152	Smith et al, 2019 [4]	NA
WDR81 + SYNJ1	Smith et al, 2019 [4]	NA
LONP1 + ATP7B	Smith et al, 2019 [4]	NA
CEP57 + DYNC2H1	Pezzani et al, 2020 [34]	+
SLC26A2 + IFT27	Rosina et al, 2022 [35]	-
DNAI1 + TJP2	Rosina et al, 2022 [35]	+
DNAI1 + TJP2	Rosina et al, 2022 [35]	-
DCLRE1C + ATM	Rosina et al, 2022 [35]	+
EVC2 + TMC1	Umair et al, 2017 [37]	+

Multiple Molecular Diagnoses (AR + AR + _)	Submitter	Consanguinidade
PXDN + NIPAL4 + OCTN2	Balci et al, 2017 [3]	+
CAPN3 + MUSK + NAV2 + ZC4H2	Herman et al, 2021 [2]	+
ACBD6 + ACCN2 + FRMD7	Mitani et al, 2021 [12]	+
PNKP + RTN2 + VRK3	Mitani et al, 2021 [12]	+
MRPS25 + PPP1R15A + PTCHD2	Mitani et al, 2021 [12]	+
JRK + KDM5A + VPS28	Mitani et al, 2021 [12]	+
SLC7A1 + VSTM2L + WARS2	Mitani et al, 2021 [12]	+
EXOSC3 + MYH1 + SMARCA1	Mitani et al, 2021 [12]	+
LPAR6 + PARD3B + PDZD2	Mitani et al, 2021 [12]	+
RANBP3L + RNASEH2A + TTLL11	Mitani et al, 2021 [12]	+
RANBP3L + APTX + TTLL11	Mitani et al, 2021 [12]	+
CLP1 + GIN1 + SLC25A45	Mitani et al, 2021 [12]	+
CNTNAP2 + STOML1 + STON2	Mitani et al, 2021 [12]	+
FBN1 + PLOD2 + TF	Farajzadeh et al, 2020 [36]	+
GBE1 + AP4M1 + TAF9B	Pehlivan et al, 2019 [11]	-
COL6A3 + ABCA7 + ADNP	Pehlivan et al, 2019 [11]	+
KLHL7 + HOXA11 + TNRC6C	Pehlivan et al, 2019 [11]	+
DRG1 + TANC1 + BRWD3	Pehlivan et al, 2019 [11]	+
BSCL2 + NDUFS3 + HIKESHI	Smith et al, 2019 [4]	NA
NSUN2 + HCF1 + VPS13B	Smith et al, 2019 [4]	NA
UBE3B + EHMT1 + KIAA1033	Smith et al, 2019 [4]	NA
PDE6A + SH3TC2 + SLC26A2	Smith et al, 2019 [4]	NA

NA = data not avaliable

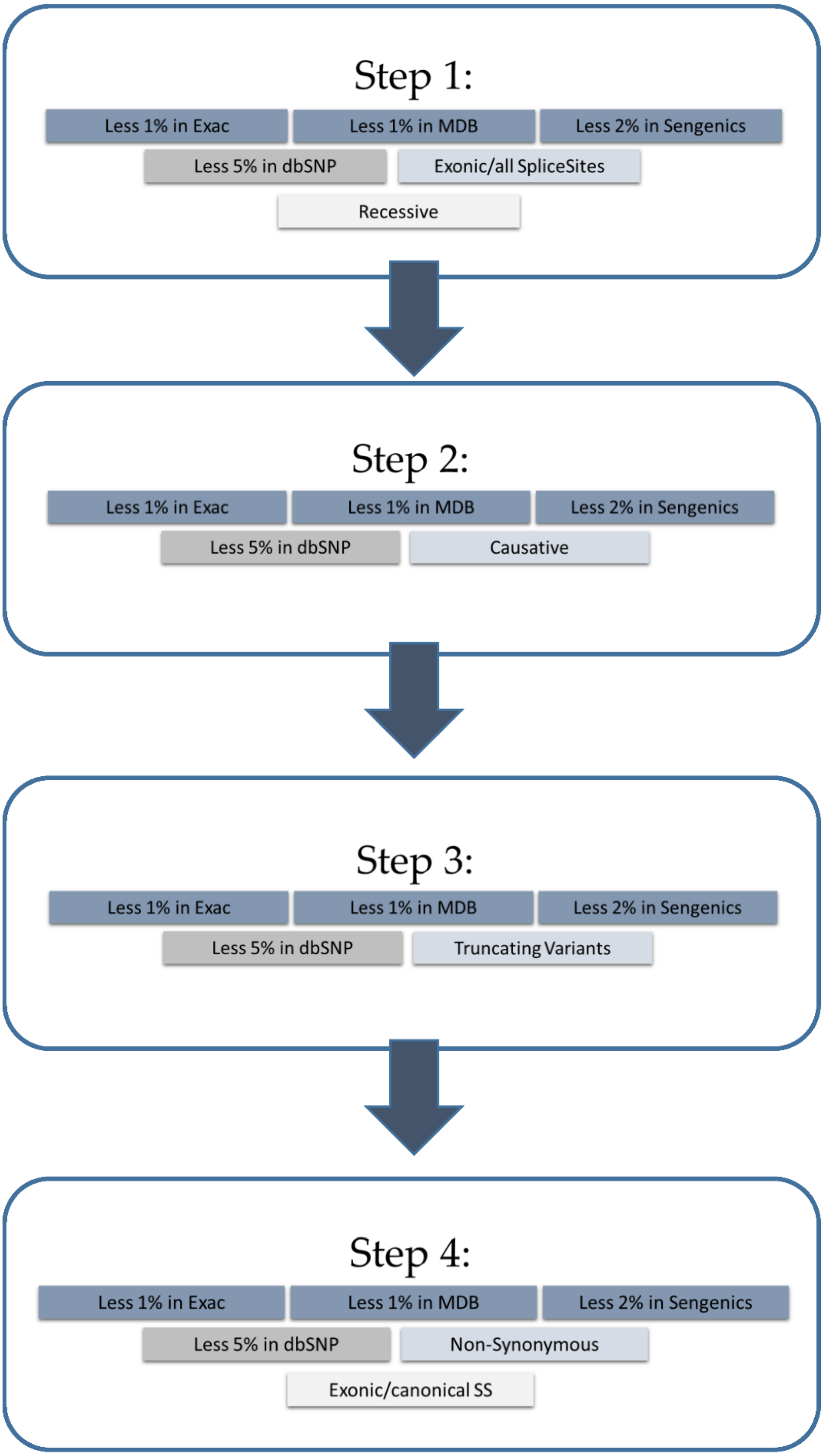

Figure S1. Flow sheet of the WES filtration steps.
